# Impact of central adiposity on the heart rate and calorie estimation accuracy of diverse smartwatches and the resulting subjective exercise experience

**DOI:** 10.3389/fspor.2026.1829700

**Published:** 2026-06-03

**Authors:** Areenta Lertyongphati, Warunya Udomkhwamsuk, Nathakorn Amornrungrojanakul, Kanakorn Suk-ieam, Wimonrat Khamyan, Phinyo Chaengchit, Warisara Asawaponwiput, Polathep Vichitkunakorn, Decho Surangsrirat

**Affiliations:** 1 Department of Computer Engineering, Thammasat University, Bangkok, Thailand; 2 Department of Computer Engineering, Chulalongkorn University, Bangkok, Thailand; 3 Digital Healthcare Platform Innovation Group, National Science and Technology Development Agency, Pathum Thani, Thailand; 4 Department of Family and Preventive Medicine, Faculty of Medicine, Prince of Songkla University, Songkhla, Thailand; 5 Health Policy Research Center, Faculty of Medicine, Prince of Songkla University, Songkhla, Thailand

**Keywords:** central adiposity, energy expenditure, ratings of perceived exertion, smartwatch validation, wearable health technology

## Abstract

**Introduction:**

In light of the growing global trend toward health awareness, wearable technologies like smartwatches have become essential for monitoring physiological indicators such as heart rate (HR). However, their utility faces two critical challenges: a technical disparity between premium and affordable devices and a conceptual gap where objective HR may fail to capture the true subjective strain experienced by diverse populations. Consequently, this study has a dual objective: to evaluate the HR accuracy of four commercially available watches and examine how measurement variations propagate through a standardized HR-derived energy expenditure model; and to investigate the dissociation between objective HR and subjective perceived exertion (RPE) in individuals with elevated central adiposity.

**Methods:**

Forty healthy adults (n=40) participated in a 45 min multi-stage exercise protocol consisting of stretching, cycling, and running. Participants were stratified into two groups based on their waist-to-height ratio (WHtR): normal (≤0.5) and elevated (>0.5). Data were synchronized using a temporal alignment procedure, and calorie expenditure was calculated through a standardized heart-rate-based regression model to ensure fair comparisons across all devices.

**Results:**

Premium smartwatches, specifically the Apple Watch and Garmin, demonstrated superior HR precision across all activity phases, maintaining high correlations (r≥0.98) with the clinical reference. While the low-cost Xiaomi and ThaiSook watches exhibited higher HR errors during motion-intensive activities, their derived calorie expenditure estimates remained remarkably stable and consistent with the reference standard. Notably, individuals with elevated WHtR reported significantly higher Ratings of Perceived Exertion (RPE) during running and recovery phases (p<0.05), despite showing no significant difference in heart rate responsiveness compared to leaner participants.

**Conclusion:**

This study confirms that while higher-end sensors offer greater heart rate precision, affordable wearables can provide sufficient HR data to yield consistent energy expenditure estimates when using a standardized mathematical model, supporting their potential utility in large-scale health monitoring. The divergence between objective heart rate and subjective exertion in participants with central adiposity indicates that heart rate alone is an insufficient gauge of exercise intensity. Consequently, personalized weight-management programs should integrate wearable-derived metrics with perceived effort to better account for the unique physiological and psychological strain associated with higher body mass.

## 1 Introduction

Wearable technologies such as smartwatches have become increasingly popular tools for monitoring health-related data. A primary function of these devices is to support individuals in maintaining a healthy lifestyle by providing convenient, continuous monitoring of key physiological indicators, with heart rate (HR) being one of the most commonly tracked. It serves as is a central metric in wearable devices because it is a foundation for other calculated features such as calorie expenditure estimates, exercise intensity zone calculations, and recovery monitoring. This reliance is supported by Keytel et al., who demonstrated that heart rate is a robust predictor of energy expenditure across various submaximal intensities [1]. Consequently, errors in HR measurement can therefore propagate to these functions, potentially compromising the user experience and outcomes.

The psychological impact of wearable feedback is equally critical. If users perceive that wearable devices provide inaccurate or unreliable data, their trust in the device diminishes, which can negatively affect exercise motivation and long-term adherence [2]. Rupp et al. emphasized that perceived trust and usability are critical factors influencing user motivation and the continued use of fitness trackers. Rupp et al. [3] While sensor technology plays a crucial role, individual physiological characteristics such as body composition introduce an additional layer of complexity to wearable accuracy and the user experience. Waist-to-height ratio (WHtR), an easily measured indicator of central adiposity, has been strongly associated with increased cardiometabolic risk, often serving as a more sensitive predictor than BMI. Ashwell and Gibson [4] Evidence suggests that individuals with higher adiposity may experience differing perceptions of effort compared to leaner individuals [5]. A systematic review by Yu et al. observed that in overweight populations, the Rating of Perceived Exertion (RPE) often diverges from objective physiological markers such as heart rate [6]. This divergence highlights the critical need for wearable devices to deliver acceptable accuracy and equitable feedback to all users regardless of body composition. Providing reliable data helps build trust and encourages consistent use, which has the potential to promote sustained physical activity and reduce the burden of chronic disease at a population level.

The rapid expansion of the wearable market has highlighted a digital health divide, driven by significant disparities in sensor technology between global market leaders and budget-friendly devices. Fuller et al. highlighted that while accuracy is improving, significant variability remains across different devices, particularly in consumer-grade models [7]. Bent et al. identified that photoplethysmography (PPG) sensors are susceptible to motion artifacts and skin-to-sensor coupling errors, which are often mitigated better in premium hardware than in budget-friendly devices [8]. Recent studies highlight performance disparities across device tiers during “transient states” of rapid heart rate change. While generally accurate during steady-state conditions, consumer wearables—particularly budget-friendly models—exhibit increased measurement errors during rapid intensity shifts [9] and often struggle to maintain clinical-grade accuracy during vigorous activity [10]. Consequently, it remains unclear whether these budget-friendly devices offer accuracy comparable to established global brands under dynamic exercise conditions. Moreover, few studies have simultaneously investigated the technical accuracy of these devices alongside the physiological and psychological responses of users with different body compositions. Understanding this interaction is crucial, as the accuracy of the feedback loop directly impacts user trust and their long-term adherence to physical activity.

Therefore, the objective of this study is to evaluate the heart rate and HR-Derived Energy Expenditure Calculation of four smartwatches against the Polar H10 chest strap, specifically comparing global market leaders Apple Watch SE and Garmin Forerunner 245 with the affordable Xiaomi Mi Watch Lite and ThaiSook Watch, a locally developed low-cost smartwatch. Gilgen-Ammann et al. (2019) have validated the Polar H10 as a criterion measure for heart rate during field-based exercise [11]. One of the goal of this research is to demonstrate the health viability of low-cost devices, proving that affordable technology can provide actionable data comparable to premium brands. As Brewer et al. argue, leveraging accessible digital health tools is essential for bridging the health equity gap in underserved communities [12]. To address the ecological validity limitations of standard steady-state testing, we employs a comprehensive 45 min exercise protocol comprising varied intensities, including resting, stretching, cycling, and running, to assess device performance under diverse motion conditions. Furthermore, we specifically examines the influence of waist-to-height ratio (WHtR) on both sensor accuracy and the subjective exercise experience. By correlating physiological data with Ratings of Perceived Exertion (RPE), we aim to provide a holistic view of how body composition shapes both the technical reliability of wearables and the subjective perception of physical effort.

## 2 Materials and methods

### 2.1 Participants

A total of 40 healthy adults (20 males and 20 females), aged 20–59 years, participated in this study. Participants were classified into two groups according to their waist-to-height ratio (WHtR): WHtR < 0.5 and WHtR > 0.5 [13]. This classification resulted in two WHtR groups comprising 18 and 22 participants, respectively, with a comparable sex distribution across groups. Their baseline demographic and anthropometric characteristics are summarized in Table 1.

**Table 1:** Demographic and anthropometric characteristics of participants stratified by Waist-to-Height Ratio (WHtR) (*n* = 40).

Variable	Overall (*n* = 40)	WHtR < 0.5 (*n* = 18)	WHtR > 0.5 (*n* = 22)
Age (years)	33.56 ± 10.27	33.56 ± 10.27	30.82 ± 7.41
Sex (Male/Female)	20/20	10/8	12/10
Height (cm)	164.83 ± 8.68	166.06 ± 9.66	163.82 ± 7.88
Weight (kg)	70.44 ± 16.91	59.84 ± 12.01	79.11 ± 15.47
BMI (${\mathrm{kg}/m}^{2}$)	25.82 ± 5.47	21.53 ± 2.79	29.34 ± 4.54
WHtR	0.5124 ± 0.0753	0.448 ± 0.0360	0.565 ± 0.0551

WHtR was selected in preference to the body mass index (BMI) as it provides a more accurate indicator of central fat distribution [4]. This metric was prioritized based on the hypothesis that central adiposity influences both exercise physiology and the accuracy of wrist-based optical heart rate sensors. Inclusion criteria included the absence of chronic cardiovascular or respiratory diseases, no musculoskeletal injuries or surgeries within the previous six months, and the ability to perform moderate-intensity treadmill and cycling exercises while wearing wrist and chest heart-rate monitors.

The clinical trial was registered according to the WHO International Clinical Trials Registry Platform (WHO-ICTRP) at the Thai Clinical Trials Registry registry ID TCTR20250807004. This study was approved by Institutional Review Board, National Science and Technology Development Agency, Ministry of Higher Education, Science, Research and Innovation, Thailand (NIRB-011-66) and was conducted in accordance with the principles of the Declaration of Helsinki. The participants provided their written informed consent to participate in this study. All collected data from the participants were kept confidential, anonymous, and accessible only to the researchers.

### 2.2 Experimental equipment

**Figure 1:**
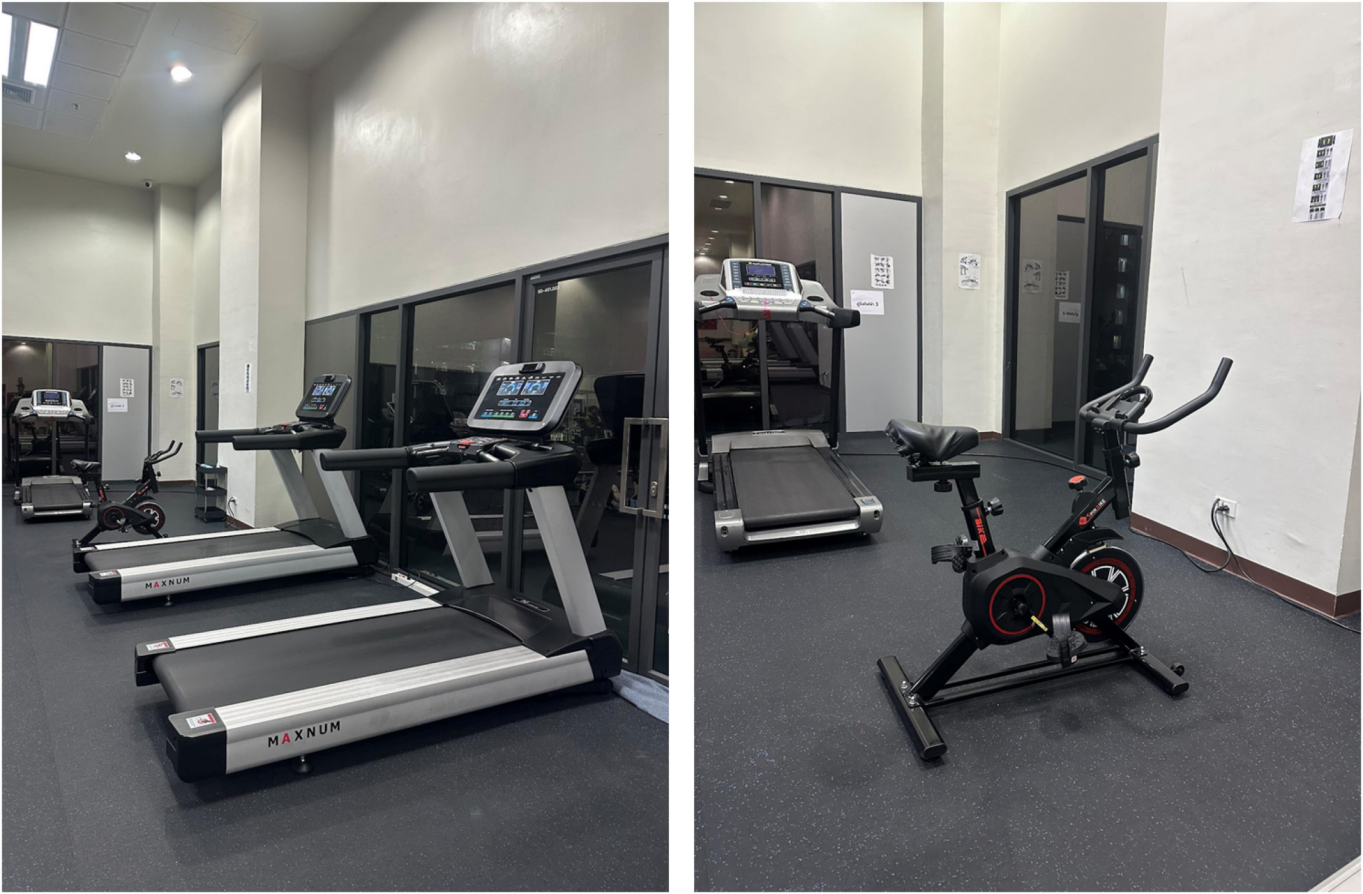
Experimental equipment used during the exercise protocol. **Left:** treadmill used for the running phase. **Right:** stationary cycle ergometer used for the cycling phase.

All exercise sessions were performed using two commercial-grade machines: a stationary cycle ergometer for the cycling phase and a treadmill for the running phase, as illustrated in Figure 1. Participants wore four commercially available wrist-worn smartwatches throughout the entire 45 min exercise protocol: ThaiSook Watch, Garmin Forerunner 245, Apple Watch SE, and Xiaomi Watch Lite. A Polar H10 chest strap heart-rate monitor served as the reference device due to its clinical-grade accuracy and high sampling frequency and as validated by Gilgen-Ammann et al. [11].

**Figure 2:**
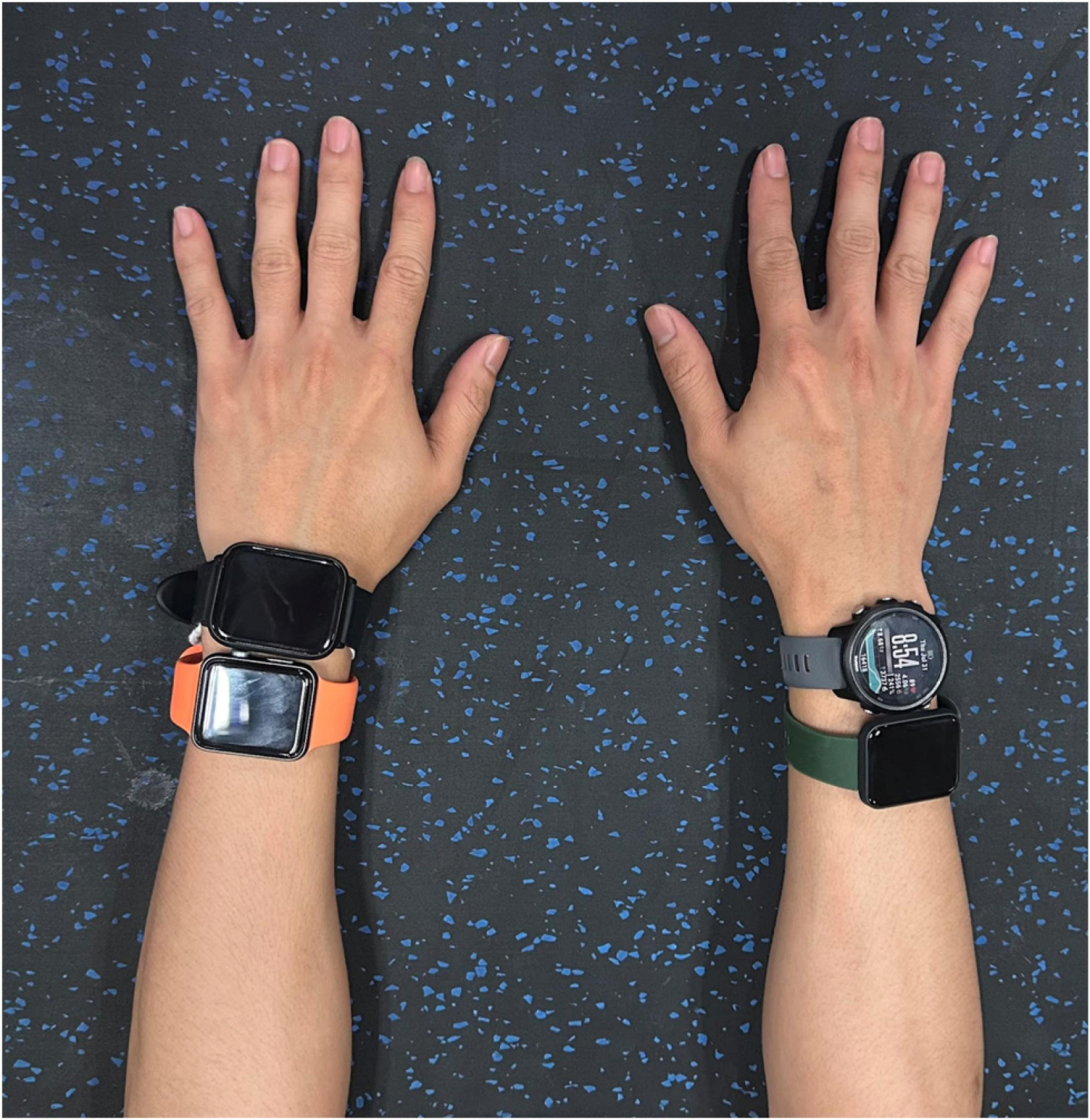
Example of smartwatch placement during the exercise protocol.

All devices were positioned according to their respective manufacturer guidelines. To ensure secure placement and to prevent accidental button contact between adjacent devices, the ThaiSook and Apple Watch were worn on the one wrist, while the Garmin and Xiaomi devices were worn on the opposite wrist. To ensure optimal and consistent skin-to-sensor coupling, participants were instructed to fasten the watch bands snugly, maintaining tightness sufficient to prevent shifting during vigorous motion without restricting blood flow. This arrangement minimized the risk of physical interference between side buttons and maintained proper spacing for continuous optical sensor contact, as shown in Figure 2.

### 2.3 Exercise protocol

**Figure 3:**
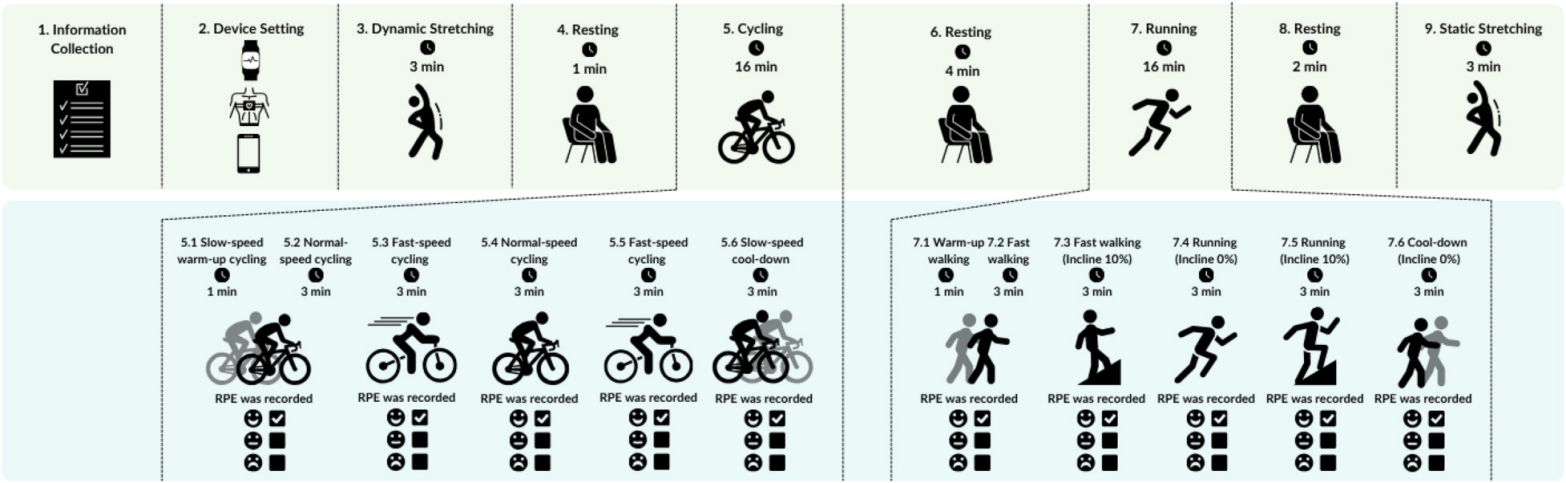
Overview of the 45 min exercise protocol, including activity types, durations, and RPE measurement points.

The study followed a structured protocol designed to assess device performance across a wide range of physiological intensities and motion conditions. As illustrated in Figure 3, the session was divided into nine distinct sequential steps:

**Step 1–2** Setup and Device Configuration: The session initiated with the collection of participant demographic data and the configuration of the wearable devices. Devices were fitted according to the placement protocol described earlier to ensure optimal sensor contact before any physical activity began. **Step 3–4: 4 min** Warm-up and Baseline Establishment: Prior to the active exercise blocks, participants performed a 3 min dynamic stretching routine to prepare the musculoskeletal system (Step 3: Dynamic Stretching). This was immediately followed by a 1 min seated resting period (Step 4: Pre-exercise Rest) to establish a physiological baseline heart rate. **Step 5: 16 min** Cycling Block: Participants completed a 16 min cycling session on a stationary bike (Step 5: Cycling) subdivided into six continuous phases to induce varying heart rate responses. The details of each segment are as follows: slow-speed warm-up (1 min); normal-speed cycling (3 min, RPE recorded); fast-speed cycling (3 min, RPE recorded); normal-speed cycling (3 min, RPE recorded); fast-speed cycling (3 min, RPE recorded); and slow-speed cool-down (3 min, RPE recorded). **Step 6: 4 min** Rest: A 4 min seated resting period (Step 6: Post-cycling Rest) was provided between the cycling and running blocks to allow heart rate recovery and assess device accuracy during recovery transitions. **Step 7: 16 min** Running Block: Participants then transitioned to a treadmill for a 16 min running session (Step 7: Running) subdivided into six continuous phases to induce varying heart rate responses. The details of each segment are as follows: warm-up walking at 0% incline (1 min); fast walking at 0% incline (3 min, RPE recorded); fast walking at 10% incline (3 min, RPE recorded); running at 0% incline (3 min, RPE recorded); running at 10% incline (3 min, RPE recorded); and cool-down walking at 0% incline (3 min, RPE recorded). **Step 8–9: 5 min** Cool-down: The protocol concluded with a 2 min seated resting period (Step 8: Post-running Rest) followed by 3 min of static stretching (Step 9: Static Stretching) to facilitate recovery.

### 2.4 Data collection

Participant demographic and anthropometric information, including age, sex, height, weight, and waist circumference, was recorded prior to the start of the exercise protocol. Heart rate data were continuously recorded from all devices throughout the entire exercise protocol. For each smartwatch and the Polar H10 chest strap, data were collected at the default sampling intervals provided by each manufacturer. At the conclusion of each exercise session, data from all devices were exported using their respective mobile applications for further processing.

**Figure 4:**
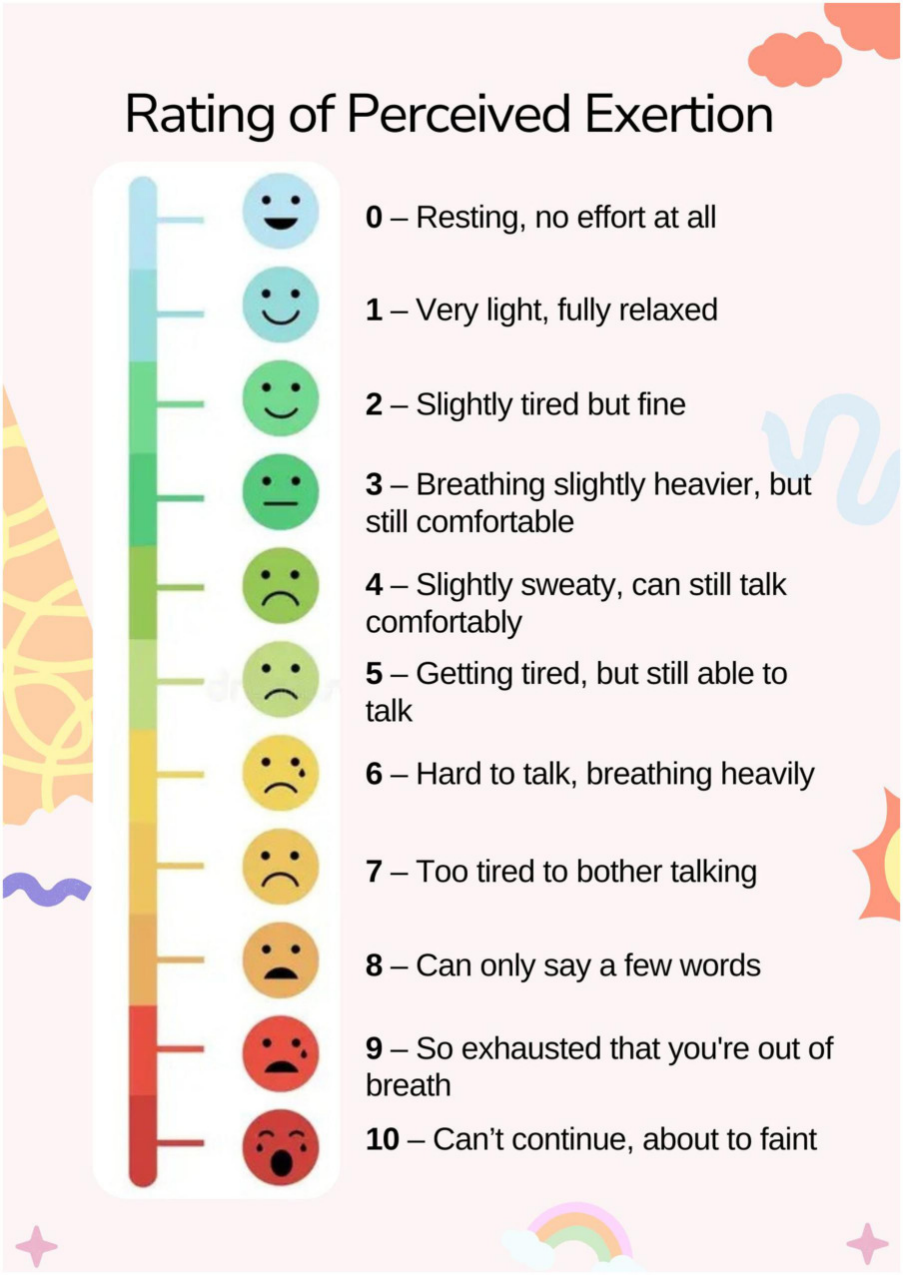
RPE (0–10) scale used during the cycling and running phases to quantify participants’ subjective perception of exertion.

In addition to heart rate data, ratings of perceived exertion (RPE) were manually collected at predefined intervals during the cycling and running phases, as described in Section 2.3. At each time point, participants were shown the RPE scale and asked to verbally indicate their rating, which was immediately recorded by the research team on standardized record sheets. These assessments were conducted using the 0–10 RPE scale illustrated in Figure 4 [14].

### 2.5 Data preprocessing

#### 2.5.1 Signal preparation and interpolation

Heart rate data from each smartwatch and the Polar H10 chest strap were exported after each exercise session. The Polar H10 served as the reference device for temporal synchronization to a common 45 min timeline. It recorded continuously at a fixed sampling rate of 1 Hz, providing a stable time base for alignment. In contrast, the commercial smartwatch devices used in this research recorded at lower or irregular sampling frequencies. The Apple Watch SE and Garmin Forerunner 245 measured heart rate at non-uniform intervals, typically every few seconds, the ThaiSook Watch produced readings at fixed 2 s intervals, and the Xiaomi Watch Lite captured data approximately once per minute. To enable consistent comparison across devices, all heart rate time series were resampled and linearly interpolated to a uniform 1 s interval. This preprocessing step standardized the sampling frequency and ensured temporal consistency with the Polar H10 reference timeline, allowing point-to-point comparison across the entire exercise protocol. While linear interpolation allows for mathematical comparison, it cannot recover missing dynamic data. Consequently, devices with sparse sampling intervals, such as the Xiaomi Watch Lite, inherently smooth over rapid transient heart rate changes. As a result, the reported error metrics for low-frequency devices during highly dynamic activity phases reflect both sensor inaccuracy and the mathematical limitation of interpolating prolonged gaps between actual sensor readings.

#### 2.5.2 Temporal alignment across devices

**Figure 5:**
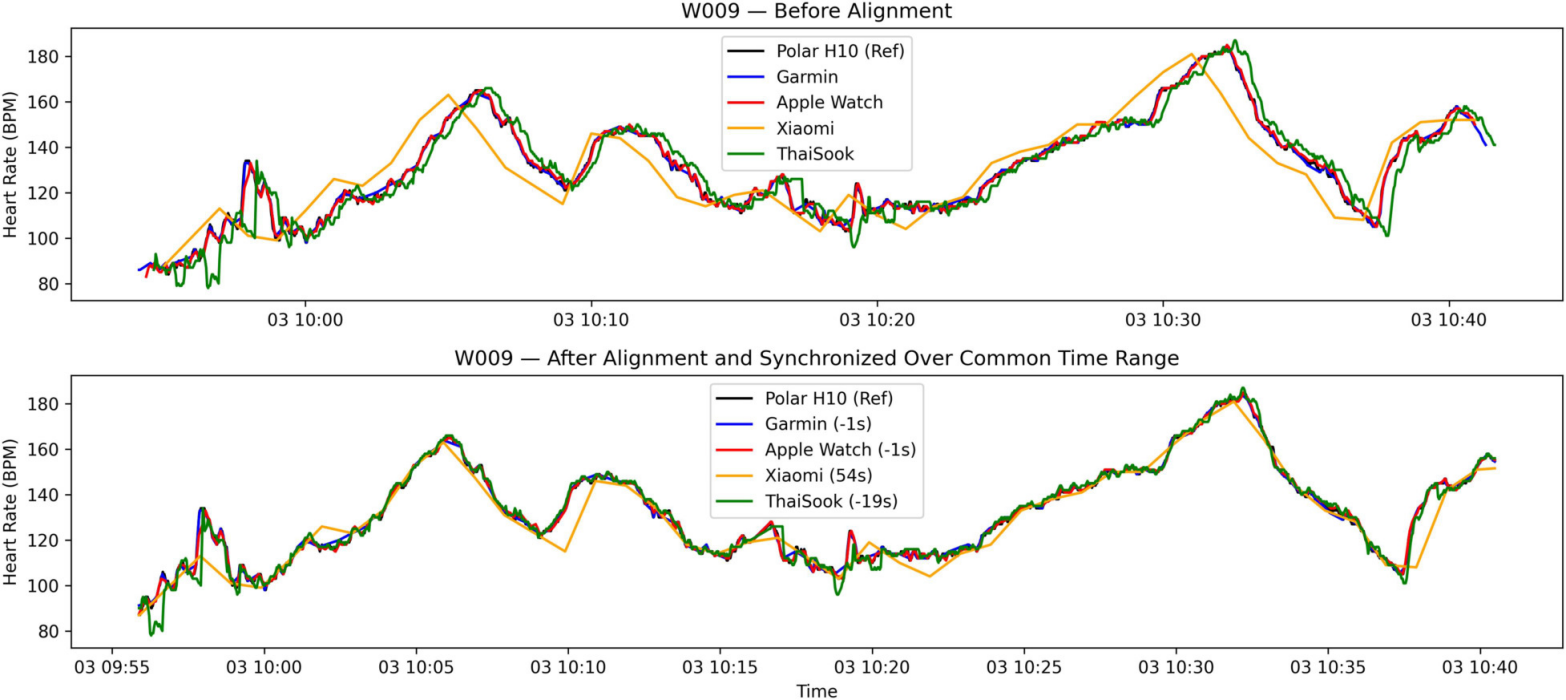
Example of heart-rate signal alignment for participant W009. The upper panel shows desynchronized smartwatch readings relative to the Polar H10 reference, while the lower panel illustrates improved synchronization after applying optimal time shifts and trimming to the common time range.

Because each smartwatch operated on an independent internal clock, it was not possible to start all five devices simultaneously at the exact same moment. In practice, the recordings were initiated manually, introducing small reaction-time differences between devices. The Polar H10 chest strap was always started last and used as the temporal baseline for all subsequent synchronization. Moreover, each device maintained its own internal timestamping system, which did not correspond exactly to real-world time due to hardware-specific delays and processing latencies. Consequently, small but consistent temporal offsets appeared across devices, as illustrated in Figure 5. In the Before Alignment plot, noticeable time lags are visible, particularly for Xiaomi and ThaiSook, resulting in phase offsets between the heart rate curves.

To correct these discrepancies, a temporal alignment procedure was applied to synchronize all smartwatch signals with the Polar H10 reference timeline. For each participant, the Polar H10 chest strap was used as the reference signal. The heart rate time series from each smartwatch was iteratively shifted within a ±300 s search window to identify the temporal offset that minimized the mean absolute error (MAE) relative to the Polar H10 reference. The alignment was implemented using a sliding-window approach that compared interpolated signals second by second. For each possible shift, the MAE was computed as:

$$\text{MAE}=\frac{1}{N}\sum_{i=1}^{N}\left|HR_{\text{ref}}(t_{i})-HR_{\text{device}}(t_{i}+\Delta t)\right|$$

where $HR_{\text{ref}}(t_{i})$ is the heart rate measured by the Polar H10 at time $t_{i}$, $HR_{\text{device}}(t_{i}+\Delta t)$ is the device signal shifted by Δt seconds, and N is the number of valid paired samples. The shift Δt yielding the smallest MAE was selected as the optimal alignment offset.

After applying the optimal time shift to each smartwatch signal, all devices were resynchronized over their common time range, ensuring that every time series started and ended simultaneously. This intersection step removed any residual temporal gaps between devices and guaranteed one-to-one correspondence of data points across the entire recording period. The After Alignment plot in Figure 5 shows tightly overlapping traces, confirming successful synchronization. This alignment was necessary to correct for human-induced manual start-time delays. However, we acknowledge that this procedure also inadvertently removes inherent hardware processing lags, effectively treating algorithmic lag as temporal noise rather than real-time error.

#### 2.5.3 Standardized HR-derived energy expenditure calculation

Calorie expenditure was independently estimated using a standardized heart rate-based model, rather than relying on the proprietary calorie outputs reported by each smartwatch. This method was selected to ensure scientific reproducibility, as proprietary algorithms are often undisclosed and subject to unannounced firmware updates. By applying a uniform equation, we evaluated all devices under identical computational criteria, effectively isolating raw sensor performance from brand-specific algorithmic assumptions. Heart rate data from all devices, including the Polar H10 chest strap and the four smartwatches, were used as inputs to the same formula for consistency. The Polar H10 served as the reference device due to its high sampling rate and clinical-grade accuracy, and its computed calorie output was treated as the ground truth for evaluating smartwatch estimates.

Energy expenditure (EE) in kilojoules per minute was estimated using the predictive equation developed by Keytel et al. [1]. This formulation, derived directly from the validated mixed-model regression, allows calorie estimation based solely on measurable physiological variables without requiring maximal oxygen uptake (VO_2max_) data. The model calculates energy expenditure (EE) in kilojoules per minute as a linear combination of heart rate (H), body weight (W), and age (A), adjusted by gender (G=1 for men, G=0 for women):

$$\begin{array}{rl} \mathrm{EE}=\; & G(-55.0969+0.6309H+0.1988W+0.2017A)\\ & +(1-G)(-20.4022+0.4472H-0.1263W+0.074A) \end{array}$$

To express this in kilocalories over a given duration T (in minutes), the equation was multiplied by T and divided by 4.184 to convert from kilojoules to kilocalories:

$$\mathrm{CB}=T\times \frac{\mathrm{EE}}{4.184}$$

#### 2.5.4 Artifact detection and data quality filtering

During visual inspection of the synchronized heart-rate signals, occasional abrupt drops to near-zero values were observed in some wrist-worn devices. These events were present in the raw recordings and are characteristic of transient photoplethysmography (PPG) signal loss, commonly caused by loose device contact, wrist motion, or poor skin coupling during exercise. Such values are physiologically implausible in healthy adults and do not represent true heart-rate measurements. To address this issue, heart-rate samples outside physiologically plausible limits were treated as invalid and excluded from analysis. Specifically, values below 30 bpm or above 220 bpm were flagged as artifacts and removed on a per-sample basis. This filtering step was applied after temporal alignment and synchronization, ensuring that all devices were evaluated on a common time base [15]. No interpolation or smoothing was applied to replace invalid samples in the quantitative analysis. All accuracy metrics were computed using only paired time points where valid heart-rate measurements were available from the smartwatch under evaluation and the Polar H10 reference device. As a result, the number of valid samples differed slightly across devices, reflecting device-specific signal dropouts during motion-intensive activities. For visualization purposes only, short signal gaps resulting from artifact removal were linearly interpolated to improve plot readability; however, all reported numerical results were derived exclusively from non-interpolated data.

### 2.6 Data analysis

The statistical framework was organized into three primary phases: validation of heart rate sensor accuracy, assessment of calorie estimation reliability, and subgroup comparisons to isolate biological variables. This comprehensive approach ensured that data was evaluated across both temporal and physiological dimensions. All analyses were performed in Python (version 3.10) using the SciPy and Pingouin packages.

#### 2.6.1 Accuracy evaluation of heart rate measurements

Accuracy was quantified using three complementary statistical metrics: mean absolute error (MAE), root mean square error (RMSE), and Pearson’s correlation coefficient (r) [16]. Each metric was computed for every smartwatch with the Polar H10 as a reference. These metrics provide complementary perspectives on average deviation, variance in error magnitude, and the linear consistency of measurements. All analyses were conducted for the full 45 min protocol and separately for each activity phase to evaluate phase-specific performance. Agreement between smartwatch and reference signals was further examined using Bland–Altman analysis, which quantified systematic bias and the 95% limits of agreement (LoA) across the full HR range [17].

To assess potential physiological influences on optical sensor accuracy, participants were divided by waist-to-height ratio (WHtR ≤ 0.5 vs. >0.5). Comparisons of MAE, RMSE, and correlation coefficients between WHtR groups were performed to determine whether body composition affected HR measurement reliability. No sex-based analysis was included, as photoplethysmography (PPG) performance primarily depends on tissue optical properties and wrist motion rather than sex-related factors.

#### 2.6.2 Consistency of standardized HR-derived energy expenditure

The consistency of calorie expenditure estimates for each smartwatch was evaluated against the Polar H10 reference using the standardized regression model described in Section 2.5.3. To ensure strict comparability, this estimation model was applied uniformly across all devices. Performance was assessed using the same statistical metrics employed for heart rate analysis; mean absolute error (MAE), root mean square error (RMSE), and Pearson’s correlation coefficient (r). These metrics were computed for both the comprehensive 45 min session and individual activity phases.

#### 2.6.3 Subgroup statistical analysis

Subgroup analyses were conducted to examine whether biological factors influenced physiological and psychological responses during the exercise protocol. These analyses focused on the measures of heart rate, percentage increase in heart rate during exercise, total calorie expenditure, and ratings of perceived exertion (RPE). Data for these comparisons were drawn strictly from the Polar H10 reference to isolate physiological variability from the technical discrepancies inherent in consumer wearables. Comparisons were performed across sex (male vs. female) and waist-to-height ratio (WHtR ≤ 0.5 vs. > 0.5) subgroups. Independent-samples *t*-tests were used to evaluate group differences, with effect sizes quantified using Cohen’s d. Statistical significance was set at α=0.05. Descriptive statistics (mean ± SD) were reported for all outcomes.

## 3 Results

### 3.1 Assessment of smartwatch heart rate measurement accuracy

The evaluation of heart rate (HR) measurement accuracy revealed that premium smartwatches maintained superior precision compared to low-cost alternatives throughout the 45 min protocol. As detailed in Table 2, the Apple Watch SE and Garmin Forerunner 55 exhibited the highest levels of agreement with the Polar H10 reference, showing consistently low Mean Absolute Error (MAE) and high Pearson’s correlation coefficients (r≥0.98 for the full session). In contrast, the budget-friendly Xiaomi Mi Watch Lite and ThaiSook Watch displayed significantly higher error rates, particularly during motion-intensive segments with Pearson’s correlation coefficients more than r≥0.84 for the full session.

**Table 2:** Accuracy metrics (Mean ± SD) for heart-rate estimation from each smartwatch compared to the Polar H10 chest strap across activity phases.

Activity phase	Device	MAE (bpm)	RMSE (bpm)	Pearson’s r
Dynamic stretching (3 min)	Apple Watch	3.35 ± 2.67	4.84 ± 3.46	0.931 ± 0.077
	**Garmin**	**3.25 ± 5.74**	**3.96 ± 6.19**	**0.959 ± 0.081**
	ThaiSook	6.93 ± 4.60	9.68 ± 5.86	0.734 ± 0.273
	Xiaomi	9.95 ± 6.34	12.61 ± 7.63	0.508 ± 0.519
Rest 1: Pre-exercise (1 min)	**Apple Watch**	**1.62 ± 1.35**	**2.21 ± 1.79**	**0.922 ± 0.130**
	Garmin	2.93 ± 5.71	3.47 ± 5.99	0.848 ± 0.312
	ThaiSook	7.16 ± 9.49	8.65 ± 9.81	0.539 ± 0.550
	Xiaomi	9.30 ± 9.88	10.42 ± 10.12	0.500 ± 0.566
Cycling (16 min)	**Apple Watch**	**1.05 ± 0.97**	**1.58 ± 1.32**	**0.977 ± 0.045**
	Garmin	1.88 ± 2.29	2.61 ± 3.35	0.930 ± 0.164
	ThaiSook	8.52 ± 11.50	11.84 ± 12.44	0.723 ± 0.295
	Xiaomi	6.96 ± 11.16	9.09 ± 12.46	0.670 ± 0.471
Rest 2: Post-cycling (4 min)	**Apple Watch**	**1.52 ± 0.98**	**2.26 ± 1.44**	**0.908 ± 0.136**
	Garmin	1.84 ± 1.37	2.50 ± 2.00	0.872 ± 0.222
	ThaiSook	5.50 ± 6.64	7.36 ± 7.32	0.556 ± 0.383
	Xiaomi	5.28 ± 4.91	6.57 ± 5.28	0.533 ± 0.411
Running (16 min)	**Apple Watch**	**1.72 ± 1.60**	**2.68 ± 2.44**	**0.987 ± 0.022**
	Garmin	2.04 ± 3.96	3.09 ± 5.55	0.982 ± 0.051
	ThaiSook	9.35 ± 7.64	12.90 ± 9.29	0.862 ± 0.122
	Xiaomi	6.80 ± 7.62	9.36 ± 10.00	0.877 ± 0.206
Rest 3: Post-running (2 min)	**Apple Watch**	**0.86 ± 0.39**	**1.22 ± 0.56**	**0.954 ± 0.126**
	Garmin	1.37 ± 0.98	1.87 ± 1.37	0.908 ± 0.187
	ThaiSook	4.86 ± 4.82	6.59 ± 5.91	0.613 ± 0.442
	Xiaomi	4.82 ± 7.25	6.04 ± 7.58	0.651 ± 0.450
Static stretching (3 min)	**Apple Watch**	**1.35 ± 1.66**	**1.84 ± 1.89**	**0.927 ± 0.128**
	Garmin	3.15 ± 5.96	3.71 ± 6.18	0.835 ± 0.369
	ThaiSook	13.91 ± 13.74	15.73 ± 13.87	0.285 ± 0.562
	Xiaomi	7.39 ± 8.53	8.69 ± 9.06	0.526 ± 0.460
Total (full session)	**Apple Watch**	**1.52 ± 1.08**	**2.69 ± 1.68**	**0.989 ± 0.017**
	Garmin	2.10 ± 2.82	3.29 ± 4.36	0.980 ± 0.041
	ThaiSook	8.60 ± 7.12	13.29 ± 8.48	0.845 ± 0.121
	Xiaomi	6.93 ± 6.91	10.45 ± 9.02	0.869 ± 0.174

Lower MAE/RMSE and higher r indicate better agreement with the reference.

Bold values indicate the smartwatch with the best agreement with the Polar H10 reference within each activity phase, based on lower MAE/RMSE and higher Pearson's *r* values.

**Figure 6:**
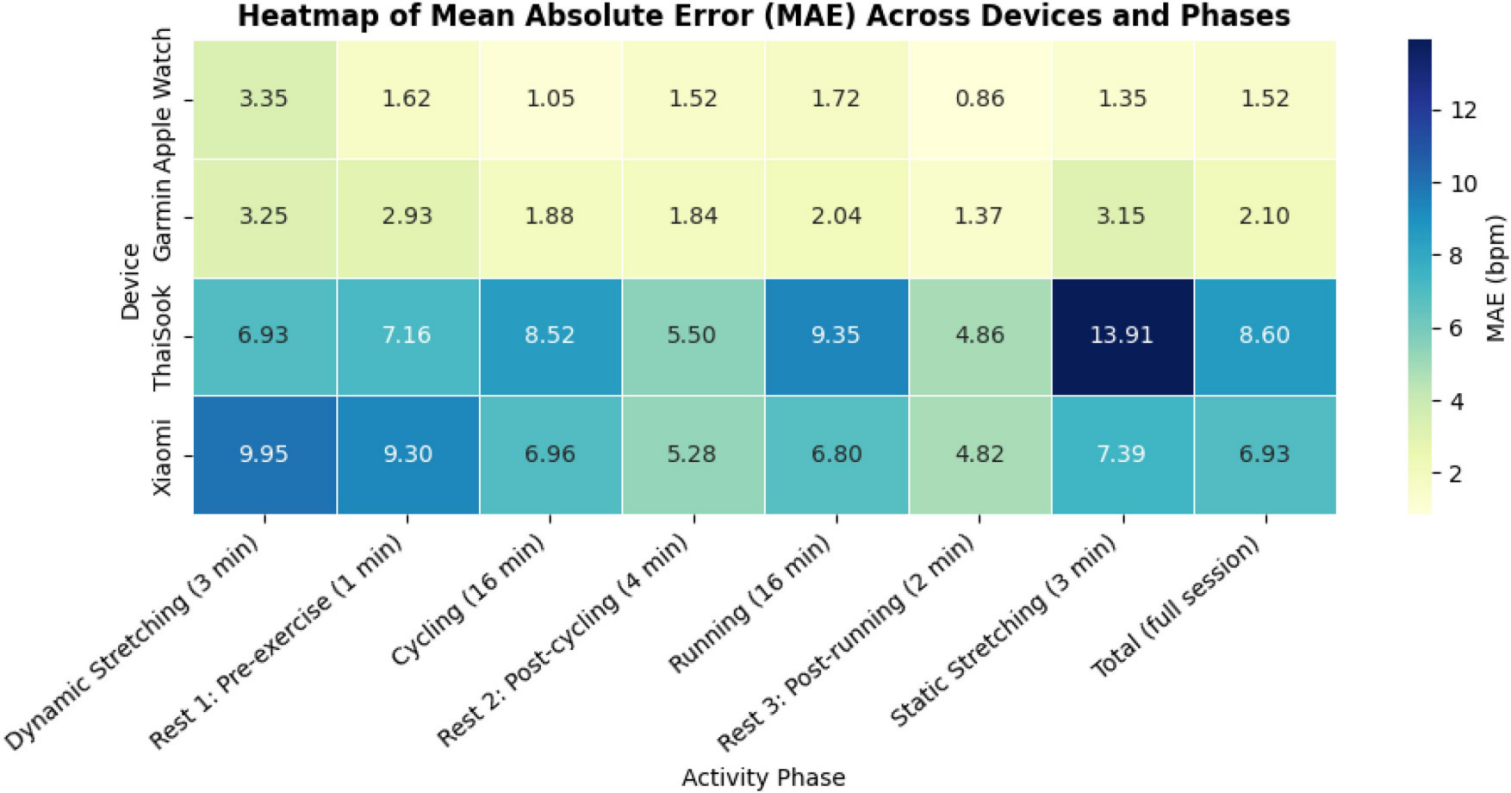
Heatmap of Mean Absolute Error (MAE) across all devices and activity phases. Cooler colors indicate lower MAE values, illustrating consistently lower errors for Apple Watch across most phases and strong performance from Garmin, particularly during dynamic stretching.

The performance disparity across activity phases is further illustrated by the MAE Heatmap in Figure 6. The heatmap visually confirms that the Apple Watch maintained the most stable performance, indicated by cooler colors across all nine stages, including a remarkably low MAE of 0.86 bpm during post-running recovery. While Garmin also performed strongly, especially during dynamic stretching (3.25 bpm), the low-cost devices showed a distinct shift toward warmer colors in the heatmap, signaling increased measurement error during transitions and high-intensity activities. This phase-specific analysis highlights that while premium sensors offer reliable tracking for clinical-grade precision, affordable wearables face challenges with signal stability during varied physical motions.

**Figure 7:**
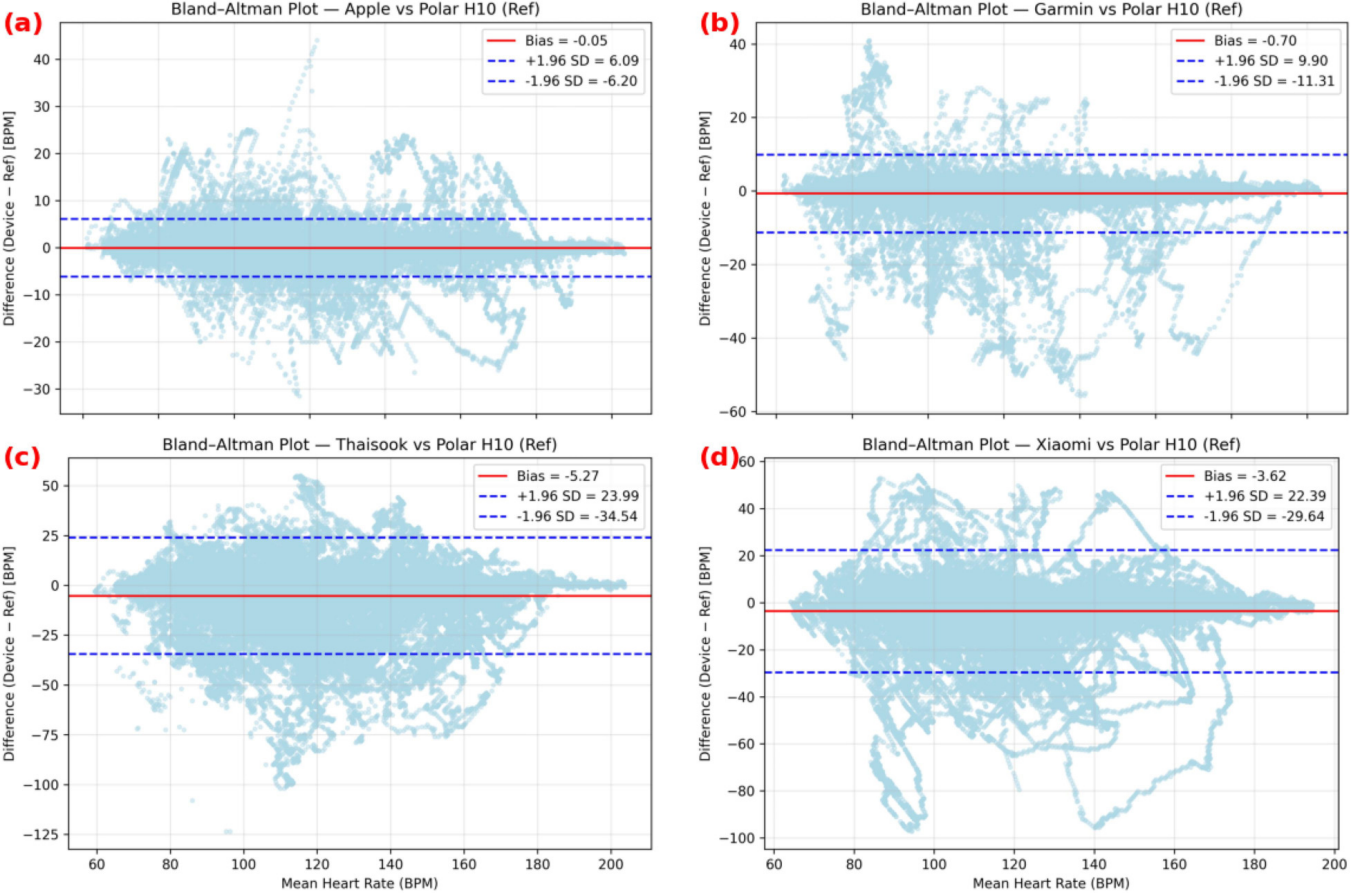
Bland–Altman plots comparing heart rate measurements from four smartwatches against the Polar H10 reference. **(a)** Apple Watch shows negligible bias (−0.05 BPM) with tight limits of agreement (−6.20 to 6.09 BPM), indicating strong agreement. **(b)** Garmin smartwatch demonstrates a small bias (−0.70 BPM) and moderate limits of agreement (−11.31 to 9.90 BPM), suggesting stable performance across heart-rate ranges. **(c)** ThaiSook smartwatch exhibits a larger negative bias (−5.27 BPM) with wide limits of agreement (−34.54 to 23.99 BPM), reflecting substantial variability and sensitivity to motion artifacts. **(d)** Xiaomi smartwatch shows an underestimation tendency (−3.62 BPM) and broad limits of agreement (−29.64 to 22.39 BPM), particularly at higher heart rates.

To further evaluate the agreement and systematic bias between the smartwatches and the clinical reference across the entire heart rate spectrum, a Bland-Altman analysis was conducted. The resulting plots, presented in Figure 7, confirm the superior accuracy of the premium devices. The Apple Watch SE demonstrated strong agreement with the Polar H10, showing a negligible mean bias of −0.05 BPM and narrow 95% limits of agreement (LoA) ranging from −6.20 to 6.09 BPM. Similarly, the Garmin Forerunner 55 exhibited stable performance across varying exercise intensities, evidenced by a small bias of −0.70 BPM and moderate LoA from −11.31 to 9.90 BPM. Conversely, the affordable alternatives displayed wider error margins and a pronounced tendency to underestimate heart rate. The ThaiSook Watch recorded a larger negative bias of −5.27 BPM with wide LoA (−34.54 to 23.99 BPM), highlighting its substantial variability and susceptibility to motion artifacts during physical activity. The Xiaomi Mi Watch Lite also showed a distinct underestimation trend, yielding a bias of −3.62 BPM and broad LoA (−29.64 to 22.39 BPM), a discrepancy that became particularly evident at higher heart rates.

#### 3.1.1 Consistency of standardized HR-derived energy expenditure

**Figure 8:**
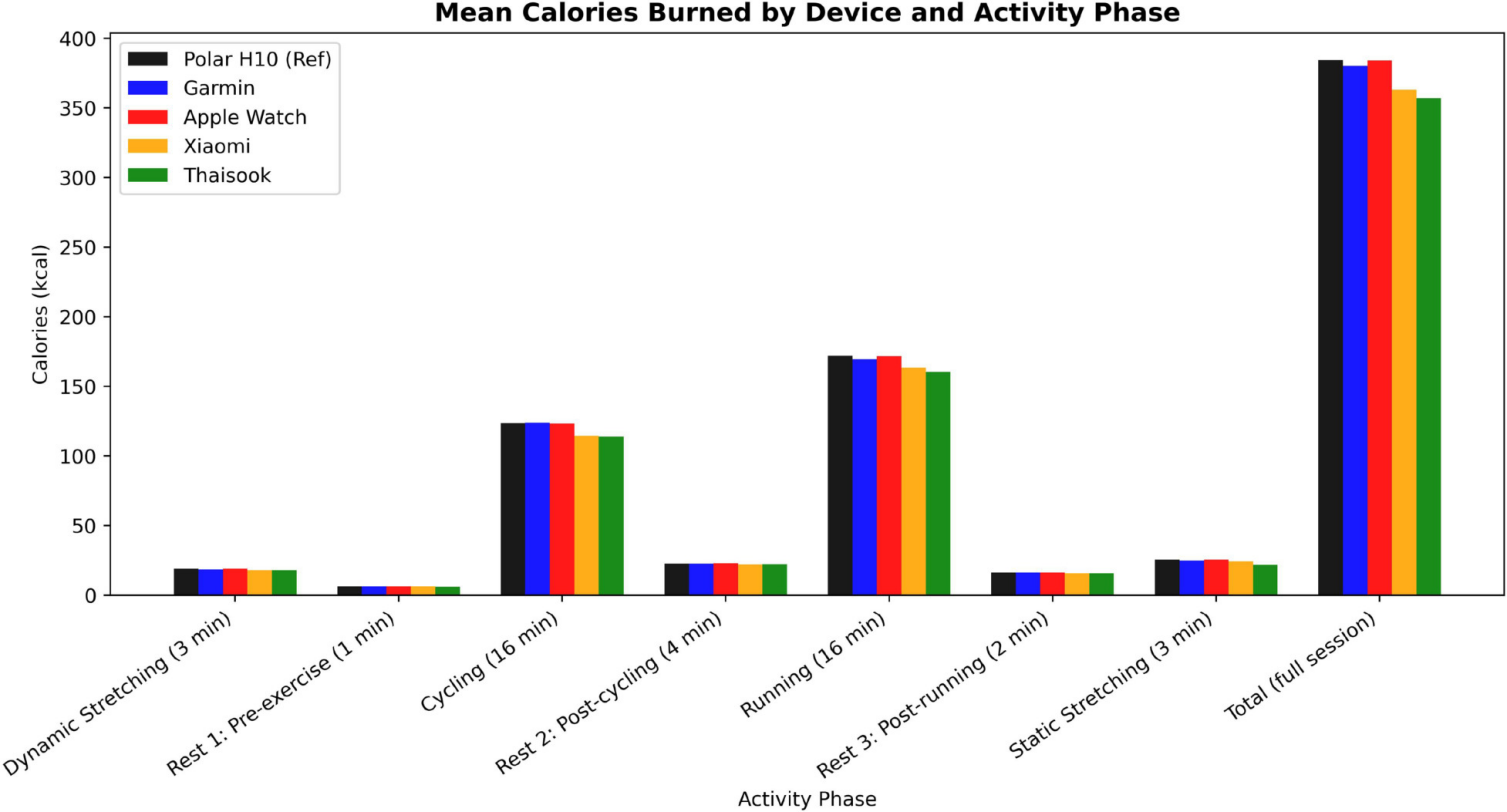
Mean calories burned by each device across activity phases.

Despite the discrepancies observed in heart rate measurement during motion-intensive activities, the derived calorie expenditure estimates remained remarkably stable and consistent with the clinical reference standard across all devices. As illustrated in Figure 8, the mean calories burned tracked closely together across all nine activity phases, regardless of the smartwatch tier. During the most demanding segments, such as the 16 min cycling and running blocks (which included intensity variations such as 0% and 10% inclines), both the premium and budget-friendly wearables produced total energy expenditure values that closely mirrored the Polar H10 baseline. This overall consistency indicates that while transient heart rate errors may occur at the sensor level, the resulting calorie estimates are smoothed over time, yielding consistent HR-derived energy expenditure estimates across devices when applying a shared mathematical model.

### 3.2 Influence of WHtR and sex on physiological and psychological responses

To isolate physiological variability from the technical discrepancies inherent in consumer wearables, baseline physiological and psychological responses from the Polar H10 reference device were analyzed across sex and waist-to-height ratio (WHtR) subgroups. As detailed in Table 3, an analysis of total energy expenditure revealed a significant difference between sexes, with male participants exhibiting a substantially higher total calorie expenditure (460.63 ± 106.09 kcal) compared to females (321.80 ± 59.71 kcal) (p<0.001, Cohen’s d=1.593). However, the average heart rate measured throughout the session did not significantly differ between males (121.36 ± 14.67 bpm) and females (125.56 ± 11.43 bpm) (p=0.3362). Similarly, when comparing participants based on body composition, neither mean heart rate nor total calorie expenditure demonstrated statistically significant differences between the normal (WHtR≤0.5) and elevated (WHtR>0.5) central adiposity groups.Cardiovascular responsiveness was further assessed by examining the percentage increase in heart rate from rest to exercise, as presented in Table 4. Transitioning from rest to cycling resulted in a 9.77 ± 16.91 percent increase for males and a 12.81 ± 15.56 percent increase for females, indicating no significant statistical difference (p=0.5592). The transition from rest to running produced a more pronounced heart rate elevation across all demographics, yet the differences remained non-significant between sexes (37.76 ± 20.11 percent for males vs. 29.90 ± 16.00 percent for females; p=0.1833). When examining WHtR, leaner individuals (WHtR≤0.5) displayed a higher mean percentage increase during the rest-to-running transition (40.08 ± 21.27 percent) compared to those with elevated adiposity (28.36 ± 13.74 percent), though this divergence approached but did not reach statistical significance (p=0.0533).

**Table 3:** Mean heart rate and total calorie expenditure by sex and waist-to-height ratio (WHtR) with statistical comparison.

Factor	Variable	(Mean ± SD)	(Mean ± SD)	*t*-value	*p*-value	Cohen’s d
Sex		Male	Female			
	Heart rate (bpm)	121.36±14.67	125.56±11.43	−0.98	0.336	−0.31
	Calories burned (kcal)	460.63±106.09	321.80±59.71	4.90	** <0.001 **	**1.59**
WHtR		≤0.5	>0.5			
	Heart rate (bpm)	120.96±12.32	125.69±13.57	−1.13	0.268	−0.35
	Calories burned (kcal)	376.63±87.04	396.84±124.42	−0.59	0.560	−0.18

Bold values denote statistical significance (*p* < 0.001), and Cohen's d > 0.8 indicates a large effect size.

**Table 4:** Percentage increase in heart rate from rest to exercise by Sex and Waist-to-Height Ratio (WHtR).

Factor	Activity	(Mean ± SD)	(Mean ± SD)	*t*-value	*p*-value	Cohen’s d
Sex		Male	Female			
	Rest to cycling	9.77±16.91	12.81±15.56	−0.59	0.559	−0.19
	Rest to running	37.76±20.11	29.90±16.00	1.36	0.183	0.44
WHtR		≤0.5	>0.5			
	Rest to cycling	14.61±18.56	8.71±13.59	1.12	0.270	0.37
	Rest to running	40.08±21.27	28.36±13.74	2.02	0.053	0.67

#### 3.2.1 Heart rate sensor accuracy by body composition

To determine whether central adiposity influences the technical reliability of wrist-worn optical sensors, heart rate accuracy metrics were further stratified by waist-to-height ratio (WHtR). As presented in Table 5, the premium devices demonstrated consistently high precision across both body composition groups. Interestingly, both the Apple Watch and Garmin Forerunner 245 recorded slightly lower Mean Absolute Error (MAE) and Root Mean Square Error (RMSE) in the elevated adiposity group (WHtR>0.5) compared to the leaner group (WHtR≤0.5). Specifically, the Apple Watch yielded an MAE of 1.362±0.805 bpm for WHtR>0.5 vs. 1.706±1.342 bpm for WHtR≤0.5. Similarly, the Garmin device showed an MAE of 1.574±1.669 bpm for the elevated WHtR group compared to 2.759±3.752 bpm for the leaner group. Among the budget-friendly alternatives, the Xiaomi Mi Watch Lite also exhibited marginally better accuracy in participants with WHtR>0.5 (MAE of 6.237±7.014 bpm) than in those with WHtR≤0.5 (MAE of 7.696±6.482 bpm). The ThaiSook Watch maintained relatively consistent, albeit higher, error rates across both demographics, with an MAE of 8.651±8.612 bpm for WHtR>0.5 and 8.777±4.946 bpm for WHtR≤0.5. Overall, these findings indicate that increased central adiposity did not negatively impact the performance of the photoplethysmography (PPG) sensors. In fact, signal reliability was comparable or even slightly superior in the higher WHtR cohort across the tested smartwatches.

**Table 5:** Heart rate accuracy metrics (mean ± SD) by WHtR group and device.

Device	WHtR group	MAE (bpm)	RMSE (bpm)	Pearson’s r
Apple	>0.5	1.36 ± 0.81	2.49 ± 1.44	0.991 ± 0.009
	≤0.5	1.71 ± 1.34	2.92 ± 1.94	0.987 ± 0.023
Garmin	>0.5	1.57 ± 1.67	2.58 ± 3.20	0.988 ± 0.026
	≤0.5	2.76 ± 3.75	4.17 ± 5.43	0.971 ± 0.054
ThaiSook	>0.5	8.65 ± 8.61	13.19 ± 10.20	0.830 ± 0.150
	≤0.5	8.78 ± 4.95	13.91 ± 5.82	0.853 ± 0.077
Xiaomi	>0.5	6.24 ± 7.01	9.43 ± 9.23	0.888 ± 0.159
	≤0.5	7.70 ± 6.48	11.65 ± 8.53	0.846 ± 0.191

#### 3.2.2 Subjective ratings of perceived exertion (RPE) between subgroup

The physiological strain reported by participants, quantified via the Rating of Perceived Exertion (RPE), revealed distinct patterns based on biological factors across different exercise modalities.

During the cycling phase, as detailed in Table 6, central adiposity (WHtR) did not significantly impact perceived exertion at any time point. However, significant differences were observed between sexes. Female participants reported significantly higher RPE than males during the second normal-speed interval at 14 min (4.00±1.30 vs. 2.53±1.71; p=0.0046, Cohen’s d=−0.98) and during the final cool-down at 20 min (4.10±1.70 vs. 2.84±1.42; p=0.016, Cohen’s d=−0.80).

**Table 6:** Cycling perceived exertion (RPE) by sex and waist-to-height ratio (WHtR) across time points.

Time (min)	Comparison	Group 1 (Mean ± SD)	Group 2 (Mean ± SD)	t-value	p-value	Cohen’s d
8 (Normal)	Sex (Male vs. Female)	2.05±1.68	3.10±1.70	−1.95	0.059	−0.62
	WHtR (≤0.5 vs. >0.5)	2.67±1.64	2.55±1.87	0.22	0.829	0.07
11 (Fast)	Sex (Male vs. Female)	3.21±2.15	4.38±1.53	−1.96	0.058	−0.63
	WHtR (≤0.5 vs. >0.5)	3.39±1.72	4.18±2.04	−1.33	0.190	−0.42
14 (Normal)	Sex (Male vs. Female)	2.53±1.71	4.00±1.30	−3.04	** 0.0046 **	−0.98
	WHtR (≤0.5 vs. >0.5)	3.06±1.43	3.50±1.85	−0.86	0.397	−0.27
17 (Fast)	Sex (Male vs. Female)	3.58±1.98	4.48±1.69	−1.53	0.134	−0.49
	WHtR (≤0.5 vs. >0.5)	3.67±1.57	4.36±2.06	−1.21	0.233	−0.38
20 (Cool-down)	Sex (Male vs. Female)	2.84±1.42	4.10±1.70	−2.53	** 0.016 **	−0.80
	WHtR (≤0.5 vs. >0.5)	3.17±1.47	3.77±1.82	−1.17	0.251	−0.36

Values are presented as mean ± SD, with corresponding t-values, p-values, and Cohen’s d.

In contrast, the running phase demonstrated a pronounced influence of waist-to-height ratio on subjective exertion, as shown in Table 7. While sex differences were not statistically significant during running, participants with elevated central adiposity (WHtR>0.5) experienced significantly higher perceived exertion compared to leaner individuals (WHtR≤0.5). This divergence was most notable during the flat running phase at 34 min (Run 0%), where the elevated WHtR group reported an RPE of 6.09±1.34 compared to 4.72±1.27 for the leaner group (p=0.002, Cohen’s d=−1.04). This elevated perception of effort persisted into the 40 min cool-down phase, with the higher WHtR group reporting significantly greater exertion (5.77±1.66 vs. 3.89±1.53; p=0.001, Cohen’s d=−1.18).

**Table 7:** Running perceived exertion (RPE) by sex and waist-to-height ratio (WHtR) across time points.

Time (min)	Comparison	Group 1 (Mean ± SD)	Group 2 (Mean ± SD)	t-value	p-value	Cohen’s d
28 (Fast walk 0%)	Sex (Male vs. Female)	2.58±1.22	3.14±1.71	−1.21	0.234	−0.38
	WHtR (≤0.5 vs. >0.5)	2.44±1.62	3.23±1.34	−1.64	0.110	−0.53
31 (Fast walk 10%)	Sex (Male vs. Female)	3.84±1.21	4.62±1.63	−1.72	0.094	−0.54
	WHtR (≤0.5 vs. >0.5)	3.78±1.56	4.64±1.33	−1.85	0.073	−0.60
34 (Run 0%)	Sex (Male vs. Female)	5.16±1.42	5.76±1.48	−1.31	0.197	−0.42
	WHtR (≤0.5 vs. >0.5)	** 4.72±1.27 **	** 6.09±1.34 **	** −3.30 **	** 0.002 **	** −1.04 **
37 (Run 10%)	Sex (Male vs. Female)	7.05±1.65	7.00±1.22	0.11	0.910	0.04
	WHtR (≤0.5 vs. >0.5)	6.61±1.29	7.36±1.47	−1.73	0.092	−0.54
40 (Cool-down)	Sex (Male vs. Female)	4.58±1.50	5.24±2.10	−1.15	0.257	−0.36
	WHtR (≤0.5 vs. >0.5)	3.89±1.53	5.77±1.66	−3.73	0.001	−1.18

Values are presented as mean ± SD, with corresponding t-values, p-values, and Cohen’s d.

**Figure 9:**
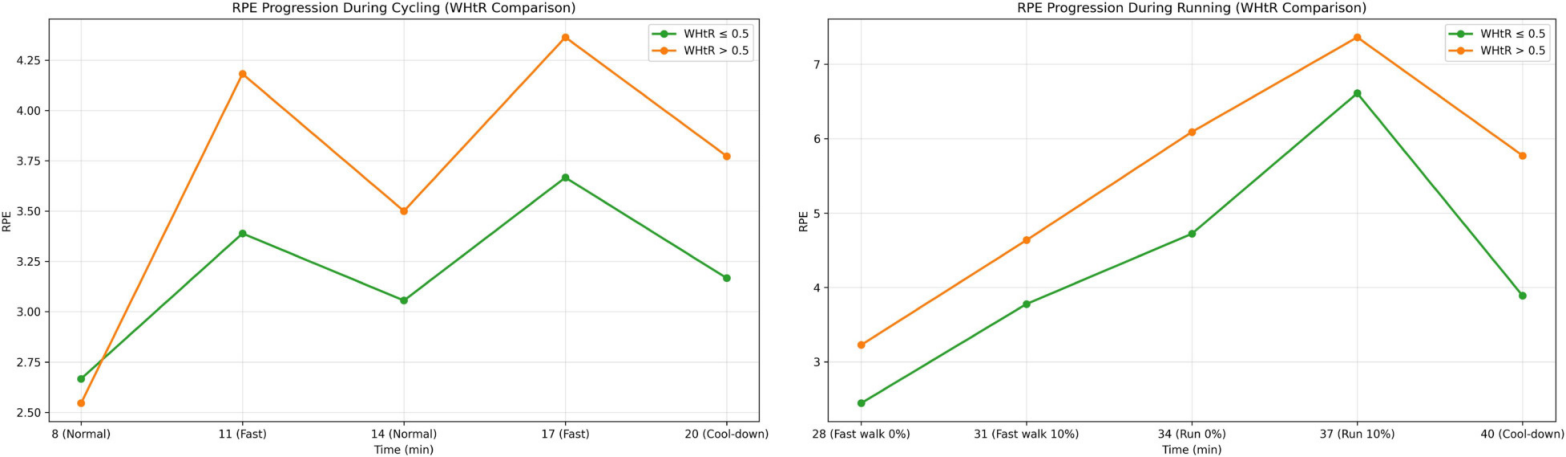
RPE progression by WHtR group during cycling and running. Mean perceived exertion (RPE) values for participants with WHtR ≤ 0.5 and WHtR > 0.5. Cycling (left) shows similar progression patterns between body composition groups, whereas running (right) highlights higher perceived exertion in the elevated WHtR group, particularly during running and recovery phases.

These subjective trajectories are visually summarized in Figure 9. The RPE progression during cycling (left panel) illustrates similar exertion patterns between the two body composition groups. Conversely, the running progression (right panel) distinctly highlights the disproportionate subjective burden experienced by the elevated WHtR group, particularly during the high-impact running and subsequent recovery phases. These findings indicate that while objective heart rate responsiveness may remain comparable across body compositions, individuals with higher central adiposity experience a significantly greater psychological and subjective burden during weight-bearing exercises like running.

## 4 Discussions

We evaluates the technical accuracy of consumer smartwatches and their psychological implications, directly addressing the utility of wearable health technologies. While a significant disparity exists in sensor technology between premium market leaders and affordable alternatives, our findings indicate that this digital divide does not fundamentally compromise energy expenditure tracking. Premium devices, specifically the Apple Watch and Garmin, maintained superior heart rate precision across all exercise phases. Conversely, the budget-friendly Xiaomi and ThaiSook watches exhibited higher error rates during motion-intensive activities. However, despite these transient heart rate discrepancies, the derived calorie expenditure estimates remained remarkably stable and consistent with the clinical reference standard across all devices.

However, our approach to evaluating energy expenditure is also our notable limitation. By applying a standardized heart rate-based regression model uniformly across all devices, we aimed to isolate sensor performance from proprietary algorithmic differences. Consequently, our findings reflect the consistency of HR-derived estimates rather than the accuracy of the commercial devices’ built-in calorie tracking algorithms. In real-world settings, the energy expenditure displayed to users on these low-cost devices may differ significantly from our standardized calculations. Moreover, by correcting for temporal lag, our reported MAE and RMSE reflect the best-case sensor fidelity under ideal synchronization. In real-world applications, the uncorrected algorithmic lag observed in low-cost devices causes delayed feedback. Consequently, the instantaneous real-time error experienced by users during rapid intensity shifts is likely higher than our aligned metrics suggest.

A critical physiological consideration in this domain is the potential for body composition, specifically skin-to-sensor coupling, to interfere with photoplethysmography (PPG) signal quality [18]. While our study standardized device placement, we did not formally quantify certain PPG confounders such as specific wrist tissue composition or use the Fitzpatrick scale for skin tone [8]. However, because our study was conducted exclusively within a Southeast Asian (Thai) cohort, the demographic homogeneity of the participants inadvertently restricted extreme variances in skin pigmentation. While this acted as a natural internal control for optical properties within our specific dataset, it also limits the generalizability of our findings. Given that higher melanin concentrations can attenuate PPG signal quality, future device validations must explicitly incorporate diverse, multi-ethnic populations to ensure equitable performance across all skin tones. Our subgroup analysis refutes the concern that thicker adipose tissue degrades measurement reliability. Increased central adiposity, defined by a waist-to-height ratio (WHtR) greater than 0.5, did not negatively impact the performance of the optical sensors. In fact, signal reliability was comparable or even slightly superior in the higher WHtR cohort across the tested smartwatches. This robust sensor performance ensures that these technologies can deliver acceptable accuracy to all users regardless of body composition, a fundamental requirement for building trust and encouraging sustained physical activity in overweight populations.

We found the significant divergence between objective physiological markers and subjective psychological strain. While cardiovascular responsiveness—measured by average heart rate and the percentage increase from rest to exercise—did not demonstrate statistically significant differences between the normal and elevated central adiposity groups, the subjective experience of effort differed entirely. Participants with elevated WHtR reported significantly higher Ratings of Perceived Exertion (RPE) during weight-bearing activities, specifically during the running and subsequent recovery phases [19].

To anchor these behavioral findings, we situate this divergence within established psychophysiological frameworks, particularly the Dual-Mode Theory of affective responses to exercise [20]. According to this framework, increasing exercise intensity is associated with a shift in the determinants of perceived exertion and affect. At lower intensities, these perceptions are primarily influenced by cognitive and environmental factors, including goals, self-efficacy, and environmental conditions. As intensity increases, interoceptive cues such as local muscular fatigue, metabolic accumulation, and cardiorespiratory strain become more dominant. This shift is most evident when physiological homeostasis is threatened, particularly at or above the ventilatory threshold.

Biomechanically, this divergence is highly task-dependent. Our data show that perceived exertion was largely similar between WHtR groups during body-weight-supported cycling Table 6, but diverged significantly during weight-bearing running Table 7. In individuals with elevated central adiposity, running imposes a substantially higher mechanical load and locomotor inefficiency, because moving excess body mass against gravity requires greater active muscle volume recruitment and increases the localized mechanical strain on the lower extremities, even when central cardiovascular responses (HR) remain comparable across groups.

Furthermore, this HR-RPE dissociation can be conceptually explained through metabolic and internal load regulation frameworks. Integrating principles from the Training-Fuel Coupling (TFC) framework, which links energy availability and substrate utilization via nutrient-sensitive signaling pathways such as AMPK–mTOR to performance optimization and adaptation, the increased mechanical work associated with higher WHtR likely elevates the local metabolic cost of locomotion [21]. The heightened muscular demand accelerates local energy depletion, shifts substrate utilization toward greater reliance on glycolytic pathways at submaximal intensities, and amplifies afferent sensory signals arising from muscle metaboreceptors and group III/IV nerve fibers. These signals are integrated in the central nervous system, contributing to a disproportionately higher internal load and a steeper rise in RPE for a given exercise intensity. Consequently, in populations with elevated central adiposity, subjective perceptual strain appears to be governed predominantly by localized muscular fatigue, locomotor inefficiency, and altered energy dynamics, rather than by central cardiovascular limits alone [22]. Recognizing this psychophysiological mechanism is important from a behavioral science perspective. A disproportionate sense of exertion, particularly during weight-bearing exercises, acts as a primary psychological barrier that directly negatively impacts exercise motivation and long-term adherence in populations with elevated adiposity.

Moving beyond psychophysiological mechanics, these findings present potential implications for the design of future digital health interventions. Current wearable ecosystems predominantly rely on standardized heart-rate zones to prescribe exercise intensity and trigger automated motivational prompts. However, our data demonstrate that for individuals with elevated central adiposity, a “moderate” objective cardiovascular zone may correspond to a “vigorous” or “near-maximal” subjective perceptual strain. If digital health platforms exclusively prompt users based on HR, they risk prescribing workloads that exceed the user’s perceptual tolerance, thereby inadvertently increasing dropout rates. To mitigate this, future digital health applications should transition from single-metric algorithms to hybrid load-monitoring systems. This could involve incorporating Ecological Momentary Assessment (EMA) directly via the smartwatch interface—prompting users to log quick RPE scores post-workout or during recovery phases [23, 24]. By utilizing these subjective inputs to help tailor motivational feedback, digital interventions can become more empathetic, sustainable, and equitably effective for populations managing weight-related challenges.

It is important to acknowledge that the generalizability of our findings is constrained by the relatively small sample size (*n* = 40), the strictly controlled laboratory setting (using stationary cycle ergometers and treadmills), and the limited demographic diversity of the cohort (healthy adults aged 20–59). Future studies should evaluate these devices in larger, more diverse populations under unstructured, free-living conditions to definitively confirm their public health impact. Finally, while the inclusion of the locally developed ThaiSook watch presents a potential conflict of interest, our use of a standardized regression model and identical statistical evaluation frameworks for all devices was specifically designed to mitigate interpretative bias and ensure objective comparisons.

## 5 Conclusion

This study confirms that while premium smartwatches offer superior precision in heart rate monitoring during dynamic exercise, affordable wearables remain highly effective for general calorie tracking. Furthermore, our findings demonstrate that central adiposity does not compromise the accuracy of optical heart rate sensors, ensuring these technologies are fundamentally reliable for populations managing weight-related challenges.

Crucially, the observed divergence between objective heart rate measurements and subjective exertion in participants with elevated central adiposity underscores that physiological metrics alone are an insufficient gauge of exercise intensity. Individuals with higher body mass experience a disproportionate psychological and physical strain during weight-bearing activities that standard heart rate zones fail to capture. Consequently, to effectively address weight-related problems and foster long-term adherence, personalized weight-management programs must evolve. Future interventions and wearable algorithms should integrate objective device-derived metrics with subjective perceived effort, ensuring a more holistic and empathetic approach to physical activity that properly accounts for the unique strain associated with higher body mass.

## Data Availability

The raw data supporting the conclusions of this article will be made available by the authors, without undue reservation.
